# Application of Metabolic ^13^C Labeling in Conjunction with High-Field Nuclear Magnetic Resonance Spectroscopy for Comparative Conformational Analysis of High Mannose-Type Oligosaccharides

**DOI:** 10.3390/biom3010108

**Published:** 2013-01-25

**Authors:** Yukiko Kamiya, Kotaro Yanagi, Toshihiko Kitajima, Takumi Yamaguchi, Yasunori Chiba, Koichi Kato

**Affiliations:** 1 Institute for Molecular Science and Okazaki Institute for Integrative Bioscience, National Institutes of Natural Sciences, 5-1 Higashiyama Myodaiji, Okazaki 444-8787, Japan; E-Mails: yukikok@mol.nagoya-u.ac.jp (Y.K.); kyanagi@ims.ac.jp (K.Y.); takumi@ims.ac.jp (T.Y.); 2 Graduate School of Pharmaceutical Sciences, Nagoya City University, 3-1 Tanabe-dori, Mizuho-ku, Nagoya 467-8603, Japan; 3 Research Center for Medical Glycoscience, National Institute of Advanced Industrial Science and Technology (AIST), Tsukuba Central 6, 1-1-1 Higashi, Tsukuba, Ibaraki 305-8566, Japan; E-Mails: kitajima@micro.biol.ethz.ch (T.K.); y-chiba@aist.go.jp (Y.C.); 4 The Glycoscience Institute, Ochanomizu University, 2-1-1 Ohtsuka, Bunkyo-ku, Tokyo 112-8610, Japan; 5 Systems and Structural Biology Center, RIKEN, 1-7-22 Suehiro, Tsurumi, Yokohama 230-0045, Japan; 6 GLYENCE Co., Ltd./2-22-8 Chikusa, Chikusa-ku, Nagoya 464-0858, Japan

**Keywords:** nuclear magnetic resonance spectroscopy, high mannose-type oligosaccharide, stable isotope labeling, *Saccharomyces cerevisiae*, nuclear Overhauser effect

## Abstract

High mannose-type oligosaccharides are enzymatically trimmed in the endoplasmic reticulum, resulting in various processing intermediates with exposed glycotopes that are recognized by a series of lectins involved in glycoprotein fate determination in cells. Although recent crystallographic data have provided the structural basis for the carbohydrate recognition of intracellular lectins, atomic information of dynamic oligosaccharide conformations is essential for a quantitative understanding of the energetics of carbohydrate–lectin interactions. Carbohydrate NMR spectroscopy is useful for characterizing such conformational dynamics, but often hampered by poor spectral resolution and lack of recombinant techniques required to produce homogeneous glycoforms. To overcome these difficulties, we have recently developed a methodology for the preparation of a homogeneous high mannose-type oligosaccharide with ^13^C labeling using a genetically engineered yeast strain. We herein successfully extended this method to result in the overexpression of ^13^C-labeled Man_9_GlcNAc_2_ (M9) with a newly engineered yeast strain with the deletion of four genes involved in *N*-glycan processing. This enabled high-field NMR analyses of ^13^C-labeled M9 in comparison with its processing product lacking the terminal mannose residue Man*D2*. Long-range NOE data indicated that the outer branches interact with the core in both glycoforms, and such foldback conformations are enhanced upon the removal of Man*D2*. The observed conformational variabilities might be significantly associated with lectins and glycan-trimming enzymes.

## 1. Introduction

The carbohydrate chains that modify proteins play key roles in a variety of physiological and pathological functions, such as intercellular communications, viral infections and immune reactions [1]. These biological processes involve molecular recognition mediated by carbohydrate–protein and carbohydrate–carbohydrate interactions, which can be potential therapeutic targets [2,3,4,5]. In addition to these extracellular events, accumulating evidence has indicated that high mannose-type oligosaccharides displayed on proteins are actively involved in glycoprotein fate determination processes in cells, such as folding, translocation and degradation, through interactions with a series of intracellular lectins [6,7,8]. In the endoplasmic reticulum (ER), a nascent polypeptide chain is covalently linked to the triantennary oligosaccharide, Glc_3_Man_9_GlcNAc_2_ (G3M9), which is subsequently trimmed by glucosidases and mannosidases to result in a variety of high mannose-type oligosaccharides. These processing intermediates exhibit specific glycotopes that are recognized by intracellular lectins functioning as molecular chaperones and cargo receptors and by those involved in ER-associated protein degradation. Namely, the glycotopes as fate determinants of glycoproteins are embedded in the triantennary structure of G3M9 and exposed with trimming by the ER enzymes.

The recently emerged crystal structures of the intracellular lectins complexed with oligosaccharides have provided atomic pictures demonstrating the mechanisms by which glycotopes are recognized by the lectins [8]. In general, however, the oligosaccharides have significant degrees of internal motions [9]. For the quantitative evaluation of the energetics of carbohydrate–lectin interactions, it is essential to understand the conformational dynamics of the oligosaccharides in their free forms, as well as in their lectin-bound forms.

NMR spectroscopy is undoubtedly a powerful method to provide atomic information on the conformations, dynamics and interactions of oligosaccharides in solution. However, NMR studies of oligosaccharide structures remain challenging compared to those of proteins, because of the poor spectral resolutions resulting from the small variations in the functional groups and the lack of a recombinant technique to produce homogenous glycoforms in sufficient quantities [10]. To overcome these difficulties, we have recently developed a methodology for the overexpression of a homogeneous high mannose-type oligosaccharide with ^13^C labeling with a genetically engineered yeast strain [11].

**Figure 1:**
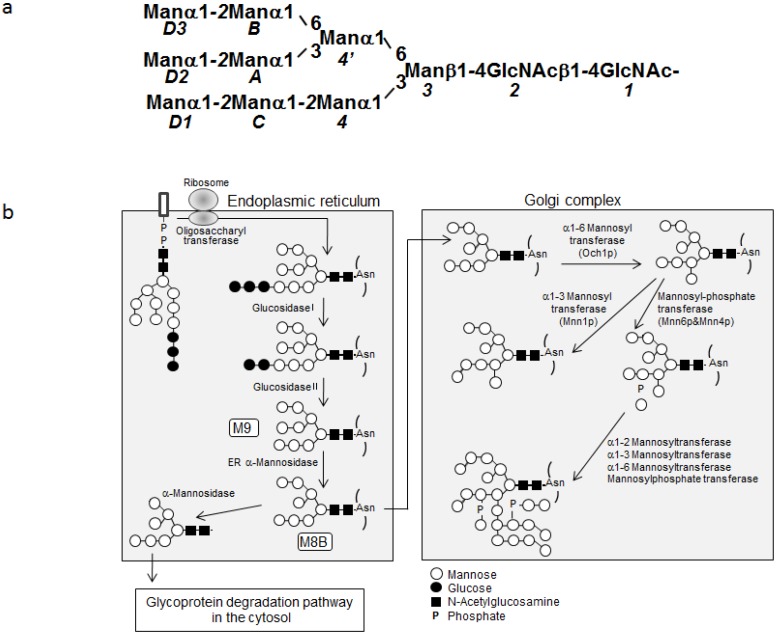
**(a)** Schematic representation of Man_9_GlcNAc_2_ showing the linkage and branching patterns, together with the residue numbering scheme. **(b)** Scheme showing the processing pathway of *N*-linked oligosaccharide (adapted from reference [11] with modifications).

In the ER of yeast, G3M9 is trimmed by glycosidases I and II, resulting in the Man_9_GlcNAc_2_ (M9) glycoform, which is then converted to the Manα1-2Manα1-6(Manα1-3)Manα1-6(Manα1-2Manα1-2Manα1-3)Manβ1-4GlcNAcβ1-4GlcNAc glycoform (abbreviated as M8B) by the removal of the terminal mannose residue (*D2*) of the central branch by the action of ER α-mannosidase [12] (Figure 1). Subsequently, glycoproteins exhibiting the M8B glycan are transported from the ER to the Golgi, where the glycan is hypermannosylated by α1-2-, α1-3- and α1-6-mannosyl transferases and phosphorylated by mannosylphosphate transferase [12]. Therefore, the M8B glycoform could exclusively accumulate in yeast by knocking out the genes encoding α1-3- and α1-6-mannosyl transferases (termed *MNN1* and *OCH1*, respectively), along with *MNN4*, which encodes a positive regulator of mannosylphosphate transferase [13,14]. By metabolic labeling with this engineered yeast strain, we have successfully prepared high yields of uniformly or site-selectively ^13^C-labeled M8B oligosaccharides of suitable homogeneity [11].

In the present study, we extended this methodology to the production of isotopically labeled M9 oligosaccharide. By comparing the NMR spectral data of ^13^C-labeled M8B and M9 collected at higher magnetic fields beyond 900 MHz of the proton observation frequency, the conformational impact of the removal of the mannose residue was examined at an atomic level.

## 2. Results

### 2.1. Overexpression of ^13^C-Labeled M9

**Figure 2:**
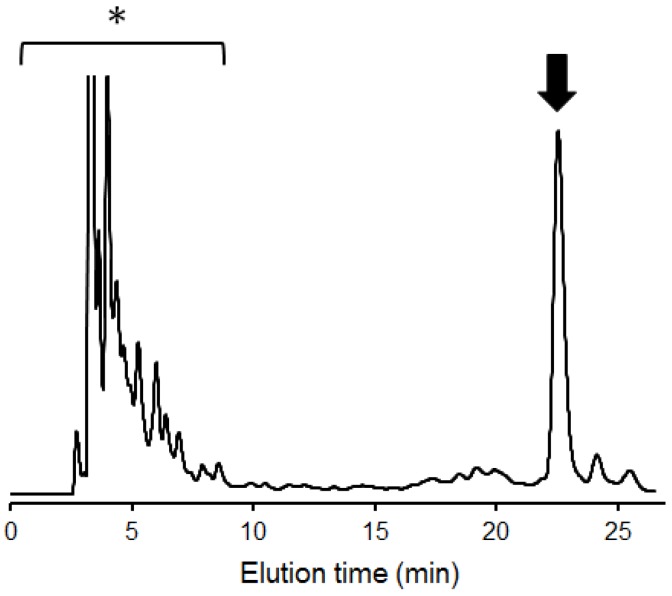
Elution profile of the pyridylamino (PA) derivative of *N*-linked oligosaccharides derived from the engineered *S. cerevisiae* cells on an Amide-80 column. The fraction indicated by the arrow corresponds to the PA derivative of Man_9_GlcNAc_2_ (M9), while those indicated by an asterisk contain no detectable oligosaccharides.

In yeast, the *N*-glycan processing pathways in the ER converge to generate the M9 glycoform, which is subsequently converted to M8B by ER α-mannosidase encoded by *MNS1*. Therefore, it was anticipated that the knocking out of this gene, in addition to *MNN1*, *MNN4* and *OCH1*, resulting in the accumulation of glycoproteins exclusively displaying M9. According to this idea, we made the Δ*och1* Δ*mnn1* Δ*mnn4* Δ*mns1* quadruple mutant of *Saccharomyces cerevisiae*. The engineered yeast cells were cultivated in a medium containing ^13^C-labeled glucose, instead of the conventional YPAD medium. From the glycoprotein mixture harvested from the cells, *N*-linked oligosaccharides were released by hydrazinolysis, re-*N*-acetylated with ^13^C-labeled acetic anhydride and subsequently fluorescence-labeled with 2-aminopyridine. The pyridylamino (PA) derivatives were subjected to high-performance liquid chromatography (HPLC), which demonstrated that the major *N*-linked oligosaccharide was identified as M9 based on the elution times (Figure 2). This result was confirmed by MS. The typical yields of PA-M9 with ^13^C labeling were 380 nmol/liter of cell culture. 

### 2.2. ^1^H-^13^C HSQC Spectral Comparison of M8B and M9

**Figure 3:**
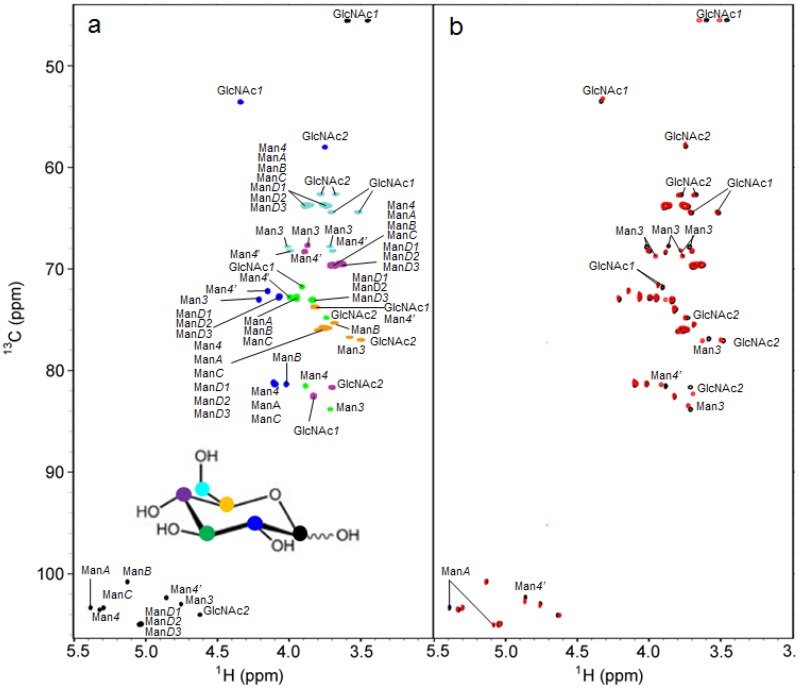
**(a)**^ 1^H-^13^C Heteronuclear Single Quantum Coherence (HSQC) spectra of the PA derivative of M9, metabolically ^13^C-labeled with D-[1-^13^C]glucose (*black*), D-[2-^13^C]glucose (*blue*), D-[3-^13^C]glucose (*green*), D-[4-^13^C]glucose (*magenta*), D-[5-^13^C]glucose (*orange*) or D-[6-^13^C]glucose (*cyan*). The six spectra were superposed and the ^13^C-labeled positions in the glucose isotopomers used as metabolic precursors are shown with circles in the same colors as the corresponding spectra. ^1^H-^13^C HSQC experiments were performed at a proton observation frequency of 920.7 MHz with 256 (*t*_1_) × 1024 (*t*_2_) complex points and 16 scans per *t*_1_ increment. The spectrum widths were 15.1 kHz for the ^13^C dimension and 5.8 kHz for the ^1^H dimension. **(b)**^ 1^H-^13^C HSQC spectra of the PA derivative of M9 (*black*) and Manα1-2Manα1-6(Manα1-3)Manα1-6(Manα1-2Manα1-2Manα1-3)Manβ1-4GlcNAcβ1-4GlcNAc glycoform (M8B) (*red*) uniformly labeled with ^13^C.

Because we already established the protocol for the preparation of ^13^C-labeled M8B, the aforementioned success in the overexpression of ^13^C-labeled M9 enabled us to perform comparative conformational analyses of these two glycoforms with stable isotope-assisted NMR methods. Figure 3 compares the ^1^H-^13^C HSQC spectra of the PA derivatives of the isotopically labeled M8B and M9 oligosaccharides. The spectral assignments were made using ^1^H-^13^C HSQC TOCSY and H2BC data in conjunction with position-selective ^13^C metabolic labeling (Figure 3a). 

The chemical shift values are summarized in Table S1. Significant chemical shift differences were observed not only in the Man*A* residue, which is exposed as the nonreducing terminus in M8B, but also in the inner core parts (Figure 3b). These data suggested that the removal of the Man*D2* residue induces overall conformational rearrangements of the triantennary structure.

We also measured one-bond ^13^C-^1^H spin-coupling constants (^1^*J*_C1,H1_) for the anomeric group of each mannose residue on the basis of *F*_2_-coupled ^1^H-^13^C HSQC spectra of the PA-derivatives of M9 and M8B metabolically ^13^C-labeled with D-[1-^13^C]glucose (Table 1). Besides Man*A*, Man*3* and Man*4’* exhibited small, but significant, differences in the ^1^*J*_C1,H1_ value between M9 and M8B. Since ^1^*J*_C1,H1_ depends on C-H bond length and orientation, as well as glycosidic linkage conformation [15,16], the observed differences again indicated some distal effects of the Man*D2* elimination.

**Table 1:** ^1^*J*_C1,H1_ values (Hz) of PA-M8B and PA-M9 metabolically labeled with D-[1-^13^C]glucose.*^ a^*

	M8B	M9
Man*3*	161.13 ± 0.02	160.86 ± 0.01
Man*4*	172.93 ± 0.05	172.77 ± 0.04
Man*C*	172.72 ± 0.05	172.70 ± 0.04
Man*D1*	*171^b^*	171.38 ± 0.02
Man*4'*	171.46 ± 0.01	171.73 ± 0.06
Man*A*	171.51 ± 0.07	173.03 ± 0.02
Man*D2*		171.26 ± 0.05
Man*B*	172.33 ± 0.03	172.24 ± 0.09
Man*D3*	*171^b^*	171.15 ± 0.08

*^a ^F*_2_*-*coupled ^1^H-^13^C HSQC experiments were performed at a proton observation frequency of 920.7 MHz with 32 (*t*_1_) × 65536 (*t*_2_) complex points and 12 scans per *t*_1_ increment. The spectral widths were 2.3 kHz for the ^13^C dimension and 2.3 kHz for the ^1^H dimension. ^1^*J*_C1,H1 _values are the mean ± S.D. of three independent experiments. *^b ^*Coupling constants that could not be determined accurately due to overlap are shown in *italics.*

### 2.3. Interresidue NOE Connectivities of M8B and M9

**Figure 4:**
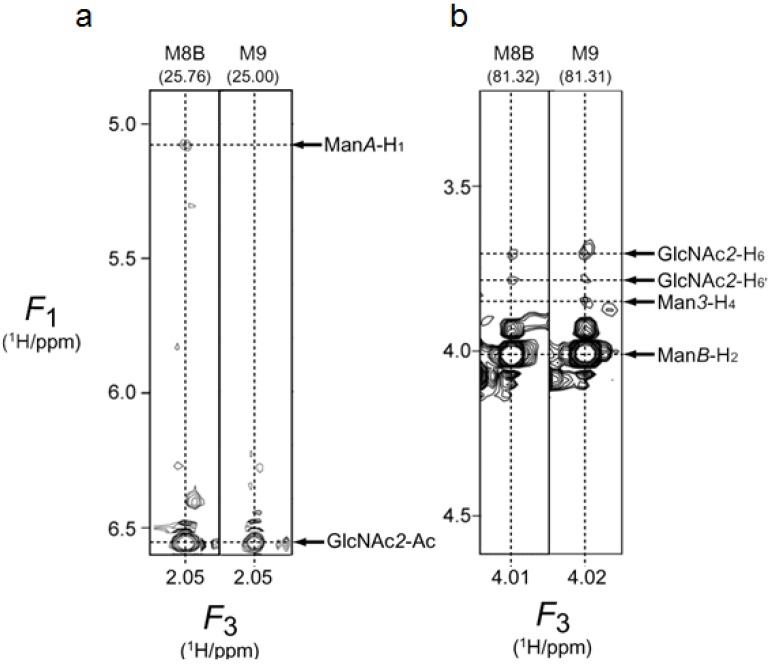
Part of the ^13^C-edited Nuclear Overhauser effect spectroscopy (NOESY) spectra of the PA derivatives of M8B and M9 uniformly labeled with ^13^C, showing connectivities from (a) GlcNAc*2*-Ac and (b) Man*B*-H_2_. The spectrum was recorded at a proton observation frequency of 950.3 MHz with 64 (*t*_1_) × 80 (*t*_2_) × 1024 (*t*_3_) complex points and two scans per *t*_1_ increment with a mixing time of 200 ms. The number at the top of each slice is the chemical shift of ^13^C resonance.

**Figure 5:**
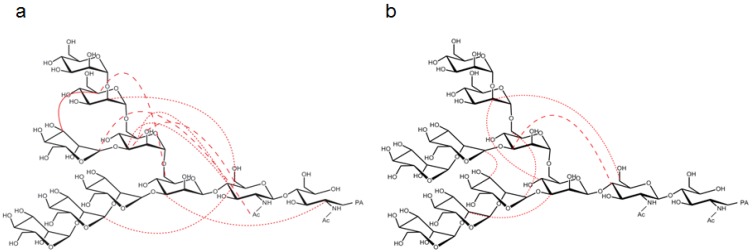
Summary of the long-range Nuclear Overhauser effect (NOE) connectivities identified in the **(a)** M8B and **(b)** M9 oligosaccharides. Interresidue NOEs are shown except for those observed between neighboring residues and those unassigned due to peak overlap. They are classified based on normalized intensity as follows: > 0.2 (solid). 0.2–0.1 (dashed), 0.1–0.03 (dotted).

In order to characterize the conformational differences between M8B and M9 with a more quantitative interpretation, we conducted ^13^C-edited NOESY experiments at a proton observation frequency of 950.3 MHz with the isotopically labeled PA oligosaccharides. As exemplified by Figure 4, not only intraresidue and interglycosidic NOEs, but also long-range NOEs, were observed exhibiting different patterns between M8B and M9. We quantified the NOE intensities by inspecting the NOE build-up curves. The results are summarized in Figure 5 and Table S2. These data clearly demonstrate that the outer branches, especially those associated with Man*4’*, are folded back toward the reducing end in both M8B and M9. Intriguingly, the NOE connectivity networks were significantly different between these two species, indicating gross conformational rearrangement of the triantennary structure upon the removal of Man*D2*, which resulted in the enhancement of the foldback conformation in M8B.

## 3. Discussion

In the present study, we successfully extended the yeast-engineering methodology to produce ^13^C-labeled M9 for detailed NMR analyses. By inspecting the ^13^C-edited NOE data, the interactions between the outer branches and the inner core were identified in M8B and M9, the former of which, however, exhibited the foldback conformation more extensively.

In their pioneering works [17,18], Vliegenthart and coworkers implied the existence of a foldback conformation in the M9 derivative lacking the innermost GlcNAc residue, based on the anomerization effects of reducing terminal GlcNAc*2* on the anomeric proton chemical shifts of Man*D2* and Man*A*, observed as their peak splitting. A more pronounced peak splitting was reported for Man*A* in the M8B derivative without GlcNAc*1*, suggesting that the removal of Man*D2* exposes Man*A*, thereby allowing its interaction with the core GlcNAc*2* residue [19]. These data are qualitatively consistent with the present NOE data obtained with the PA derivatives of M8B and M9.

The foldback conformation was also identified in the M8B glycan attached to ribonuclease B by observing a long-range NOE between Man*A*-H_1_ and GlcNAc*2*-Ac, which was also observed in the present study [20]. Similarly, the heterogeneous high mannose-type *N*-glycans[(Man)*_n_*GlcNAc_2_, *n* = 5–8] displayed on a soluble form of human CD2 (shCD2) expressed in CHO cells showed long-range NOE connectivities between Man*A*-H_1_/H_2_ and GlcNAc*2*-Ac, which, however, were not observed when Man*D2* was present [21,22]. Here, we did not detect the NOE between Man*A*-H_2_ and GlcNAc*2*-Ac in M8B (Figure 5), and this was probably because the NOE observed in shCD2 originated from its major glycoforms, such as M7 (40%) and M6 (34%) [21]. On the other hand, the undetectability of the long-range Man*A*-GlcNAc NOEs in the presence of Man*D2* was either because of the low abundance of the *D2*-containing glycoform (6%) or a conformational difference [21]. We detected no NOE between Man*D2* and GlcNAc*2* in M9 (Figure 4a), supporting a gross conformational rearrangement upon the removal of the terminal *D2* residue.

The conformational rigidity of M9 has been proposed by inspecting NMR-derived local conformational restraints, such as interglycosidic NOEs and scalar couplings [23,24] and MD simulations in explicit water [25], while *in vacuo* MD simulations have suggested considerable variabilities of the glycosidic angles of M9 [26]. In particular, 1-ns MD simulations in water showed that M9 exhibited conformational fluctuation within a restricted conformational space with maintenance of an extended overall conformation in which several strongly coordinated water molecules mediated interbranch hydrogen bonds [25]. Consistently, our ^13^C-edited NOE data identified NOE connectivities among the outer branches of M9 (Figure 5 and Table S2). However, it should be noted that the present study also indicated intramolecular interactions between the outer branch and core of M9 associated with several long-range NOE connectivities (Figure 5), which could not be interpreted solely based on the ensemble of extended conformations. Because NMR parameters, including NOE, provide an average picture of a dynamic conformational ensemble, the long-range NOE connectivities are supposed to reflect transient, minor and foldback conformations assumed by M9, which might be barely detected in the MD simulation that is conducted for a limited period. Such conformational variabilities of M9, as well as of M8B, might be significantly associated with their affinities and specificities for the intracellular lectins and glycan-trimming enzymes.

## 4. Experimental Section

### 4.1. Yeast Engineering

*S. cerevisiae* TIY19 (*MAT*a *och1*Δ::*hisG mnn1*Δ::*hisG mnn4*Δ::*hisG* *leu2-3*, *112 his3-11 ade2-1 ura3-1 trp1-1 can1-100*) was generated from TIY20 strain (*MAT*α *och1*Δ::*hisG mnn1*Δ::*hisG mnn4*Δ::*hisG leu2-3*, *112 his3-11 ade2-1 ura3-1 trp1-1 can1-100*) [27] by tetrad dissection. *MNS1* disruption was performed by a one-step PCR-mediated technique [28]. In brief, a *TRP1* cassette containing upstream and downstream sequences of the *MNS1* gene was amplified with two primers (mns1-del-f and mns1-del-r, Table 2) and the plasmid pFA6a-TRP1 as a template. The amplified DNA fragment was transformed into the TIY19 strain using a lithium acetate procedure [29], and the cells were then grown on a SDCA–Trp plate (0.67% yeast nitrogen base without amino acid, 2% glucose, 0.01% adenine hemisulfate, 0.5% casamino acids, 0.3M KCl and 2% agar). In order to identify transformants in which the *TRP1* cassette had been properly integrated, colony direct PCR using two primers (T2.1 and mns1-del-chk, Table 2) was conducted, and a PCR product of the expected size (605 bp) confirmed *MNS1* deletion of the transformants. 

**Table 2:** Primers used in this study.

Name	Sequence
mns1-del-f	CAGAAAAAGGTAGTAAAAGAGGAAAAGGTTAAACATTGAAAAAGGATTCTCGGATCCCCGGGTTAATTAA
mns1-del-r	CGCATAGTGAATTTTAAAAGGCGAATCTGGCCACTATATAGCACACTAACGAATTCGAGCTCGTTTAAAC
mns1-del-chk	AAGTGCAGAGGCGCTATC
T2.1	TCTGCAAGCCGCAAACTT

### 4.2. Metabolic Labeling

The engineered yeast cells were grown in medium containing 0.5% ^13^C-labeled D-glucose (Cambridge Isotope Laboratories, Inc. MA, USA) and 0.67% yeast nitrogen base without amino acids (Difco), supplemented with 0.3 M KCl as an osmotic stabilizer for 72 h at 30 °C. The cells were harvested by centrifugation at 5000× *g* for 10 min at 4 °C, washed with distilled water and re-centrifuged. The cells were resuspended in 4 mL of 100 mM citrate buffer (pH 7.0) per 1 g of yeast cells and subsequently lysed by autoclaving at 121 °C for 120 min, according to the literature [30]. After the collection of the supernatant by centrifugation at 8000× *g* for 10 min, three volumes of cold ethanol were added to precipitate the glycoproteins. The collected precipitates were dissolved again with water and then lyophilized for the next reaction. 

### 4.3. Purification

In order to release *N*-linked oligosaccharides, lyophilized glycoproteins (15 mg) were resolved in 0.7 mL of hydrazine anhydrous (TCI, Tokyo, Japan) in a 10 mL glass tube, incubated at 95 °C for 8 h and then quenched with 7 mL of 50 mM ammonium acetate buffer (pH 7.0). Excess hydrazine and peptides were removed with a graphite carbon column (GL-Pak Carbograph, GL Sciences Inc., Tokyo, Japan) according to the literature [31]. The oligosaccharide solutions were loaded onto the GL-Pak Carbograph column. After the column was washed with 15 mL of 50 mM ammonium acetate buffer (pH 7.0), the released oligosaccharides were eluted with 5 mL of a mixture of 50 mM ammonium acetate buffer (pH 7.0): acetonitrile (40:60) containing 2% [U-^13^C_4_] acetic anhydride (Cambridge Isotope Laboratories, Inc. MA, USA), which was used for the re-*N*-acetylation of the GlcNAc residues. The eluted oligosaccharides were fluorescently labeled with 2-aminopyridine (Wako Pure Chemical Industries, Ltd., Osaka, Japan) for further purification. The PA oligosaccharides were sequentially fractionated and isolated by HPLC on a TSK-gel Amide-80 column (Tosoh Corporation, Tokyo, Japan) and a Shim-pack HRC ODS column (Shimadzu Corporation, Kyoto, Japan), as described in the literature [32,33,34,35]. The identification of oligosaccharide structures was based on their elution position from these columns in comparison with PA-glycans in the GALAXY database [32,34]. The identification of the M8B and M9 oligosaccharides as PA derivatives were confirmed by MALDI-TOF-MS analyses using Voyager DE-STR (Applied Biosystems).

### 4.4. NMR Measurements and Analyses

Purified oligosaccharides were dissolved in 99.96% D_2_O at a concentration of 0.3 mM. NMR spectra were obtained at 303 K using a JEOL ECA-920 spectrometer or a Bruker Avance-III 950 spectrometer equipped with a cryogenic probe. Chemical shifts of ^1^H were referenced to DSS (0 ppm), while ^13^C chemical shifts were referenced indirectly, using the gyromagnetic ratios of ^13^C (γ^13^C/γ^1^H = 0.25144952). ^1^H-^13^C HSQC spectra, H2BC and ^1^H-^13^C HSQC-TOCSY were recorded at a proton observation frequency of 920.7 MHz. 3D ^13^C-edited NOESY was recorded at a proton observation frequency of 950.3 MHz with mixing times of up to 400 ms. NMR spectra were processed using NMRPipe software [36] and analyzed with SPARKY (T.D. Goddard and D.G. Kneller, SPARKY 3, University of California, San Francisco). 

## 5. Conclusions

The method of ^13^C labeling combined with high-field NMR spectroscopy has now opened new opportunities for detailed conformational analyses of oligosaccharides. In this study, we successfully observed extensive datasets of long-range NOE connectivities in M8B and M9, which will enable experimental validation of computational simulations for the characterization of dynamic conformational ensembles of these oligosaccharides. To improve the accuracy of the validation, additional experimental data are necessary as sources of long-range geometric information. In previous studies, we demonstrated that pseudocontact shifts observed by paramagnetic lanthanide tagging provide long distance information for validating the conformational ensemble of oligosaccharides derived from MD simulations [37,38]. Paramagnetic relaxation enhancement and residual dipolar coupling are also useful for characterizing the conformational dynamics of oligosaccharides [39,40]. The stable-isotope-labeling technique in conjunction with higher magnetic fields will offer a technical basis to facilitate the collection of these NMR datasets for the elucidation of the conformational dynamics of the high mannose-type oligosaccharides in their free and lectin-bound forms.

## Appendix

**Table S1:** Chemical shift values (ppm) of PA-M8B and PA-M9.

^13^C resonances			^1^H resonances			^13^C resonances			^1^H resonances		
residue	Atom	Chemical shift	residue	Atom	Chemical shift	residue	Atom	Chemical shift	residue	Atom	Chemical shift
GlcNAc*1*	1	45.66	GlcNAc*1*	1	3.51	GlcNAc*1*	1	45.60	GlcNAc*1*	1	3.46
	1'	45.65		1'	3.64		1'	45.60		1'	3.55
	2	53.11		2	4.31		2	53.22		2	4.31
	3	71.43		3	3.97		3	71.57		3	3.93
	4	82.46		4	3.83		4	82.52		4	3.82
	5	*73.86 * *^a^*		5	*3.82 *		5	*73.93 *		5	*3.82 *
	6	64.40		6	3.53		6	64.41		6	3.51
	6'	64.39		6'	3.72		6'	64.41		6'	3.70
	Ac	24.98		Ac	1.92		Ac	24.75		Ac	1.93
GlcNAc*2*	1	104.01	GlcNAc*2*	1	4.61	GlcNAc*2*	1	103.98	GlcNAc*2*	1	4.61
	2	57.73		2	3.74		2	57.86		2	3.73
	3	74.81		3	3.75		3	74.77		3	3.73
	4	82.41		4	3.67		4	81.73		4	3.68
	5	77.05		5	3.50		5	77.10		5	3.49
	6	62.73		6	3.69		6	62.76		6	3.69
	6'	62.72		6'	3.79		6'	62.76		6'	3.78
	Ac	25.76		Ac	2.05		Ac	25.00		Ac	2.05
Man*3*	1	103.10	Man*3*	1	4.76	Man*3*	1	102.98	Man*3*	1	4.75
	2	72.89		2	4.21		2	72.96		2	4.20
	3	83.47		3	3.72		3	83.83		3	3.70
	4	68.18		4	3.78		4	67.70		4	3.86
	5	77.08		5	3.63		5	76.90		5	3.58
	6	68.77		6	3.76		6	67.92		6	3.70
	6'	68.78		6'	3.95		6'	67.94		6'	4.00
Man*4*	1	103.49	Man*4*	1	5.33	Man*4*	1	103.53	Man*4*	1	5.32
	2	81.32		2	4.08		2	81.35		2	4.08
	3	72.75		3	3.99		3	72.81		3	3.98
	4	*69.64*		4	*3.69 *		4	*69.69 *		4	*3.68 *
	5	*75.94 *		5	*3.73 *		5	*75.94 *		5	*3.72 *
	6	*63.73 *		6	*3.75 *		6	*63.73 *		6	*3.74 *
	6'	*63.73 *		6'	*3.87 *		6'	*63.73 *		6'	*3.86 *
Man*C*	1	103.33	Man*C*	1	5.30	Man*C*	1	103.32	Man*C*	1	5.29
	2	81.17		2	4.10		2	81.14		2	4.10
	3	*72.82 *		3	*3.95 *		3	*72.84 *		3	*3.94 *
	4	*69.64 *		4	*3.69 *		4	*69.69 *		4	*3.68 *
	5	*75.94 *		5	*3.73 *		5	*75.94 *		5	*3.72 *
	6	*63.73 *		6	*3.75 *		6	*63.73 *		6	*3.74 *
	6'	*63.73 *		6'	*3.87 *		6'	*63.73 *		6'	*3.86 *
Man*D1*	1	104.93	Man*D1*	1	5.04	Man*D1*	1	104.95	Man*D1*	1	5.04
	2	*72.73 *		2	*4.06 *		2	*72.79 *		2	*4.06 *
	3	*73.03 *		3	*3.84 *		3	*72.95 *		3	*3.83 *
	4	*69.* *54 *		4	*3.6* *3 *		4	*69.57 *		4	*3.62 *
	5	*75.94 *		5	*3.73 *		5	*75.94 *		5	*3.72 *
	6	*63.73 *		6	*3.75 *		6	*63.73 *		6	*3.74 *
	6'	*63.73 *		6'	*3.87 *		6'	*63.73 *		6'	*3.86 *
Man*4'*	1	102.71	Man*4'*	1	4.86	Man*4'*	1	102.35	Man*4'*	1	4.85
	2	72.15		2	4.14		2	72.13		2	4.14
	3	81.39		3	3.91		3	81.54		3	3.88
	4	68.28		4	3.88		4	68.30		4	3.88
	5	*73.86 *		5	*3.82 *		5	*73.93 *		5	*3.82 *
	6	68.22		6	3.70		6	68.22		6	3.68
	6'	68.20		6'	4.00		6'	68.22		6'	3.98
Man*A*	1	105.02	Man*A*	1	5.08	Man*A*	1	103.31	Man*A*	1	5.38
	2	*72.73 *		2	*4.06 *		2	81.14		2	*4.09 *
	3	73.06		3	3.88		3	*72.* *84 *		3	*3.* *94 *
	4	*69.* *54 *		4	*3.69 *		4	*69.69 *		4	*3.68 *
	5	*75.94 *		5	*3.73 *		5	*75.94 *		5	*3.72 *
	6	*63.73 *		6	3.75		6	*63.73 *		6	*3.74 *
	6'	*63.73 *		6'	*3.87 *		6'	*63.73 *		6'	*3.86 *
Man*D2*	1		Man*D2*	1		Man*D2*	1	104.95	Man*D2*	1	5.04
	2			2			2	*72.79 *		2	*4.06 *
	3			3			3	*72.95 *		3	*3.83 *
	4			4			4	*69.57 *		4	*3.62 *
	5			5			5	*75.94 *		5	*3.72 *
	6			6			6	*63.73 *		6	*3.74 *
	6'			6'			6'	*63.73 *		6'	*3.86 *
Man*B*	1	100.77	Man*B*	1	5.14	Man*B*	1	100.75	Man*B*	1	5.13
	2	81.32		2	4.01		2	81.31		2	4.02
	3	*72.82 *		3	*3.95 *		3	*72.84 *		3	*3.94 *
	4	*69.64 *		4	*3.69 *		4	*69.69 *		4	*3.68 *
	5	75.44		5	3.68		5	75.47		5	3.68
	6	*63.73 *		6	*3.75 *		6	*63.73 *		6	*3.74 *
	6'	*63.73 *		6'	*3.87 *		6'	*63.73 *		6'	*3.86 *
Man*D3*	1	104.93	Man*D3*	1	5.04	Man*D3*	1	104.95	Man*D3*	1	5.04
	2	*72.73 *		2	*4.06 *		2	*72.79 *		2	*4.06 *
	3	*73.03 *		3	*3.84 *		3	*72.95 *		3	*3.83 *
	4	*69.* *54 *		4	*3.6* *3 *		4	*69.57 *		4	*3.62 *
	5	*75.94 *		5	*3.73 *		5	*75.94 *		5	*3.72 *
	6	*63.73 *		6	*3.75 *		6	*63.73 *		6	*3.74 *
	6'	*63.73 *		6'	*3.87 *		6'	*63.73 *		6'	*3.86 *

*^a^*Chemical shifts that could not be determined accurately due to overlap are shown in *italics.*

**Table S2:** Normalized NOE intensities of the interresidue proton pairs of PA-M8B and PA-M9*^a^*.

M8B	Normalized intensity	M9	Normalized intensity
*1*H_2_-*3*H_3_	0.10	*1*H_3_-*2*H_1_	0.05
*1*H_3_-*2*H_1_	0.12	*1*H_4_-*2*H_1_	1.29
*1*H_4_-*2*H_1_	1.86	*1*H_6_-*2*H_1_	0.26
*1*H_4_-*2*Ac	0.08	*1*H_6_-*2*Ac	0.14
*1*H_5_-*2*H_1_	0.51	*1*H_6'_-*2*H_1_	0.22
*1*H_6_-*2*H_1_	0.54	*1*H_6'_-*2*Ac	0.18
*1*H_6_-*2*Ac	0.43	*2*H_3_-*3*H_1_	0.10
*1*H_6'_-*2*H_1_	0.23	*2*H_4_-*3*H_1_	1.31
*1*H_6'_-*2*Ac	0.19	*2*H_4_-*3*H_2_	0.04
*1*Ac-*2*H_5_	0.06	*2*H_4_-*4'*H_3_	0.12
*2*H_2_-*3*H_1_	0.07	*2*H_5_-*3*H_1_	0.07
*2*H_3_-*3*H_1_	0.18	*2*H_6_-*B*H_2_	0.06
*2*H_4_-*3*H_1_	1.92	*2*H_6'_-*3*H_1_	0.16
*2*H_4_-*3*H_2_	0.08	*2*H_6'_-*3*H_2_	0.18
*2*H_4_-*4'*H_3_	0.06	*2H_6'_-3*H_3_	0.40
*2*H_4_-*4'*H_5_	0.09	*2*H_6'_-*B*H_2_	0.05
*2*H_5_-*3*H_1_	0.17	*3*H_1_-*4*H_1_	0.05
*2*H_6_-*3*H_1_	0.35	*3*H_2_-*4*H_1_	0.21
*2*H_6_-*3*H_2_	0.24	*3*H_2_-*4*H_3_	0.08
*2*H_6_-*4'*H_3_	0.19	*3*H_3_-*4*H_1_	1.84
*2*H_6_-*B*H_2_	0.09	*3*H_3_-*4*H_2_	0.31
*2*H_6_-*C*H_1_	0.05	*3*H_3_-*C*H_2_	0.08
*2*H_6'_-*3*H_1_	0.24	*3*H_4_-*4*H_1_	0.19
*2*H_6'_-*3*H_2_	0.19	*3*H_4_-*4'*H_1_	0.03
*2*H_6'_-*B*H_2_	0.06	*3*H_4_-*B*H_2_	0.08
*2*Ac-*A*H_1_	0.19	*3*H_5_-*4'*H_1_	0.10
*3*H_1_-*C*H_2_	0.11	*3*H_6_-*4'*H_1_	0.90
*3*H_1_-*4*H_1_	0.09	*3*H_6_-*4'*H_2_	0.42
*3*H_2_-*4*H_1_	0.28	*3*H_6'_-*4'*H_1_	0.32
*3*H_3_-*4*H_1_	1.72	*4*H_1_-*4'*H_6'_	0.03
*3*H_3_-*4*H_2_	0.39	*4*H_2_-*C*H_1_	1.99
*3*H_4_-*4*H_1_	0.18	*4*H_3_-*A*H_1_	0.04
*3*H_5_-*4'*H_1_	0.14	*C*H_1_-*D1*H_1_	0.11
*3*H_5_-*4*H_1_	0.09	*C*H_2_-*D1*H_1_	1.44
*3*H_6_-*4'*H_1_	1.45	*4'*H_2_-*A*H_1_	0.20
*3*H_6_-*4'*H_2_	0.11	*4'*H_3_-*A*H_1_	1.09
*3*H_6_-*B*H_5_	0.15	*4'*H_4_-*A*H_1_	0.28
*3*H_6'_-*4'*H_1_	0.46	*4'*H_4_-*B*H_1_	0.09
*4*H_2_-*C*H_1_	2.08	*4'*H_6_-*B*H_1_	0.75
*C*H_1_-*D1*H_1_	0.23	*4'*H_6'_-*A*H_1_	0.08
*C*H_2_-*D1*H_1_	1.32	*4'*H_6'_-*B*H_1_	0.34
*C*H_3_-*D1*H_1_	0.12	*A*H_1_-*D2*H_1_	0.11
*4'*H_2_-*A*H_1_	0.13	*A*H_2_-*D2*H_1_	1.44
*4'*H_3_-*A*H_1_	1.19	*B*H_1_-*D3*H_1_	0.24
*4'*H_4_-*A*H_1_	0.14	*B*H_2_-*D3*H_1_	0.84
*4'*H_6_-*B*H_1_	1.38	*B*H_3_-*D3*H_1_	0.06
*4'*H_6'_-*B*H_5_	0.49		
*A*H_3_-*B*H_5_	0.28		
*B*H_1_-*D3*H_1_	0.46		
*B*H_2_-*D3*H_1_	1.09		

*^a^*NOE intensities were normalized to the average value of the intraresidue H_1_-H_2_ NOE intensities of the mannose residues.
